# Supplementary material for: Regulation of Inflammation-Related Genes through Fosl1 Suppression in a Levetiracetam-Treated Pilocarpine-Induced Status Epilepticus Mouse Model
Source: Int J Mol Sci. 2022 Jul 9;23(14):7608. doi: 10.3390/ijms23147608 (PMC9317701; doi:10.3390/ijms23147608)
Supplement: Supplementary file 1 [file ijms-23-07608-s001.zip › ijms-1782203-supplementary.pdf]

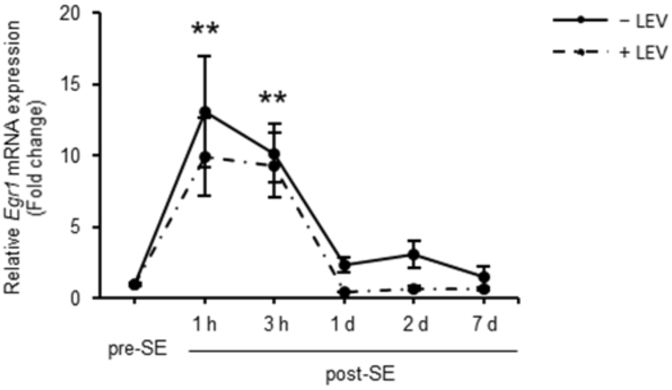


**Figure S1.** Real-time PCR analysis of *Egr1* mRNA expression in the hippocampus after SE in mice treated with LEV or untreated. Total RNA was extracted from the hippocampi of mice treated with PILO at 1 h, 3 h, 1 day, 2 days and 7 days post-SE and pre-SE in mice treated with LEV or mice that were untreated. The values were normalized relative to the β-actin (*Actb*) mRNA level and then divided by that of the control group (pre-SE) to calculate the relative mRNA levels. All data points represent the mean ± SEM of three independent experiments ($n = 3–9$). Data were analyzed for significant differences by one-way ANOVA and Tukey's post-hoc test. Results with $p$ values < 0.05 were considered statistically significant. ** $p < 0.01$ vs. pre-SE mice.
